# The Long Shadow of Repair: Late-Onset Atrioventricular Block and Atrial Arrhythmias After Scimitar Syndrome and Mitral Annuloplasty

**DOI:** 10.3390/reports8020072

**Published:** 2025-05-18

**Authors:** Fulvio Cacciapuoti, Ciro Mauro, Salvatore Crispo, Gerardo Carpinella, Mario Volpicelli

**Affiliations:** 1 Division of Cardiology, “A. Cardarelli” Hospital, 80131 Naples, Italy; 2 Department of Cardiology, Division of Arrhythmology, “Santa Maria della Pietà” Hospital, 80035 Nola, Italy

**Keywords:** scimitar syndrome, atrioventricular block, mitral annuloplasty, atrial strain, leadless pacemaker, case report

## Abstract

Background and Clinical Significance: Scimitar Syndrome is a rare congenital cardiopulmonary anomaly characterized by partial anomalous pulmonary venous return, often requiring early surgical correction. It may coexist with other congenital or acquired cardiovascular anomalies, including valvular diseases such as mitral regurgitation. When surgical correction of Scimitar Syndrome is combined with mitral valve annuloplasty, the proximity to the atrioventricular node may potentially predispose patients to late-onset conduction disturbances, although causality remains speculative. Case Presentation: We describe the case of a 53-year-old male who developed paroxysmal atrial fibrillation, atrial flutter, and intermittent second-degree AV block decades after undergoing surgical correction of Scimitar Syndrome with concomitant mitral annuloplasty. Multimodal echocardiographic evaluation revealed preserved left atrial volume, normal intra-atrial conduction time, mildly reduced strain, and maintained atrial synchrony. The patient was treated with direct oral anticoagulants and beta-blockers and underwent the implantation of a ventricular leadless pacemaker. Conclusions: This case highlights the supportive role of atrial function imaging in assessing atrial health and informing rhythm management and procedural choices in surgically corrected congenital heart disease.

## 1. Introduction and Clinical Significance

Scimitar Syndrome is a rare congenital anomaly involving partial anomalous pulmonary venous return, most commonly affecting the right lung, with drainage into the inferior vena cava [1]. It often becomes symptomatic during infancy or early childhood, and surgical correction is typically indicated to redirect pulmonary venous return to the left atrium and address associated anomalies [2]. In some cases, this correction is accompanied by mitral valve interventions such as annuloplasty to treat coexistent mitral regurgitation [3].

Mitral annuloplasty, though effective in restoring valve competence, can involve anatomical regions near the atrioventricular (AV) node and the bundle of His [4]. Although direct causation is difficult to establish in delayed presentations, this anatomical proximity raises the possibility of conduction disturbances, both immediate and late-onset [5]. Long-term sequelae such as first- or second-degree AV block may develop insidiously, years after initial surgery. Additionally, the atrial surgical substrate, coupled with age-related fibrosis, can predispose to atrial arrhythmias including atrial fibrillation (AF) and atrial flutter [6].

Recent advances in echocardiographic imaging, especially tissue Doppler imaging (TDI) and speckle-tracking echocardiography (STE), have enabled the precise evaluation of left atrial (LA) mechanics, including intra-atrial conduction time and mechanical synchrony [7]. These tools are increasingly used to guide management decisions in arrhythmia care, particularly in patients with structural heart disease or previous cardiac surgery [8,9]. LA strain, in particular, serves as a sensitive marker of atrial remodeling and functional reserve, complementing volumetric assessments [10,11].

Clinical Significance: This report presents a case of delayed AV conduction disturbances and atrial arrhythmias in a patient decades after surgical correction of Scimitar Syndrome and mitral annuloplasty. We discuss the role of echocardiographic findings, including strain imaging, in guiding therapeutic strategies and avoiding unnecessary ablation.

## 2. Case Presentation

**Figure 1:**
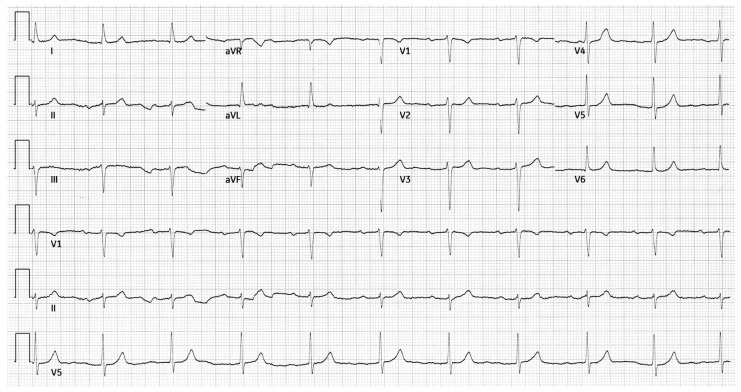
Resting 12-lead ECG showing sinus rhythm, first-degree AV block (PR interval: 248 ms), and left axis deviation.

A 53-year-old male with a history of Scimitar Syndrome presented for an evaluation of recurrent arrhythmias. At age 19, he had undergone surgical redirection of the anomalous right pulmonary vein into the left atrium. Concomitantly, he received mitral valve annuloplasty to address mild mitral regurgitation. The patient had an uneventful postoperative recovery and remained clinically stable for over two decades. Serial imaging over the years did not reveal significant changes in mitral valve function, and mitral regurgitation remained stable. In his mid-40s, the patient developed paroxysmal atrial fibrillation managed with direct oral anticoagulation, amiodarone, and beta-blockers. He subsequently developed atrial flutter, treated successfully with electrical cardioversion. Shortly thereafter, he experienced a transient Mobitz type I AV block, which resolved following the discontinuation of amiodarone. Approximately one year later, he had a syncopal episode during which a Mobitz type II AV block was documented. The conduction abnormality resolved spontaneously. The patient also referred a history of hiatal hernia and gastroesophageal reflux disease, managed conservatively. A resting 12-lead ECG showed sinus rhythm with a PR interval of 248 ms and left axis deviation (Figure 1).

**Figure 2:**
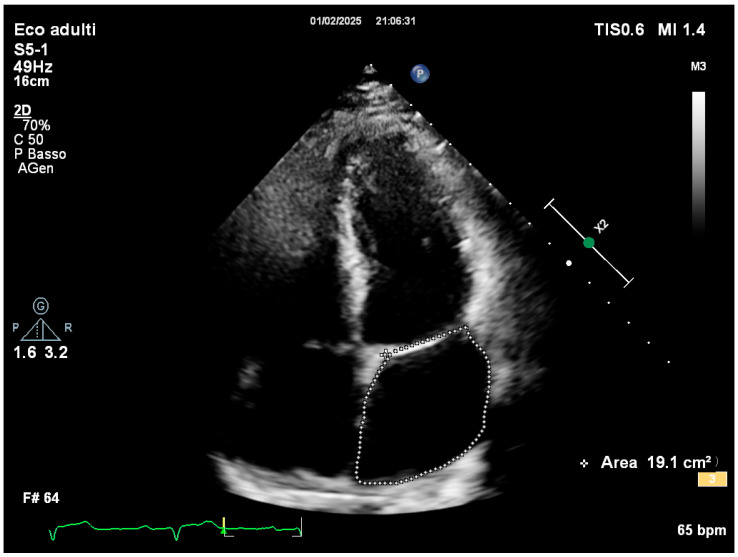
Left atrial area measured in apical 4-chamber view.

Comprehensive transthoracic echocardiographic evaluation revealed a normal left atrial volume, with no evidence of dilation and a left atrial volume of 19.1 cm^2^ (Figure 2).

**Figure 3:**
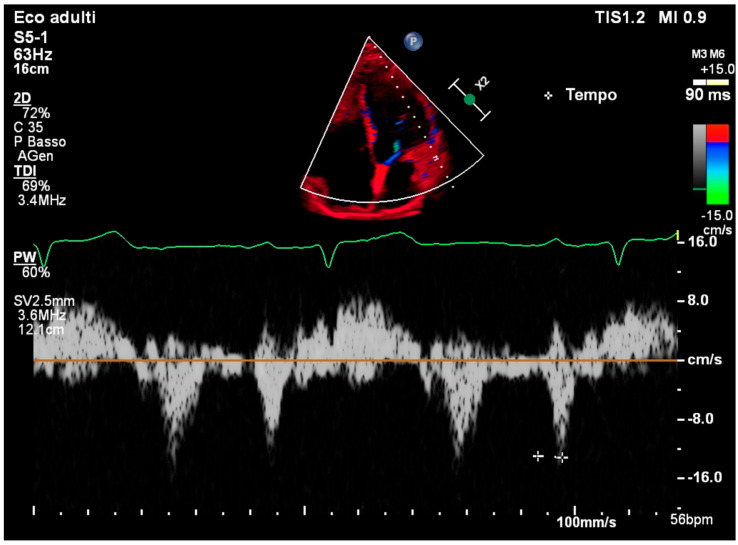
Tissue Doppler imaging of the lateral mitral annulus showing normal PA—TDI interval.

TDI of the lateral mitral annulus showed a normal PA—TDI interval of 90 ms, consistent with preserved intra-atrial conduction (Figure 3).

STE revealed mildly reduced left atrial longitudinal strain values, particularly in posterior and septal segments. Despite this, global atrial strain remained within functional thresholds. Importantly, the strain curves demonstrated preserved synchrony across atrial segments, with uniform timing of peak contraction and no evidence of mechanical dispersion.

**Figure 4:**
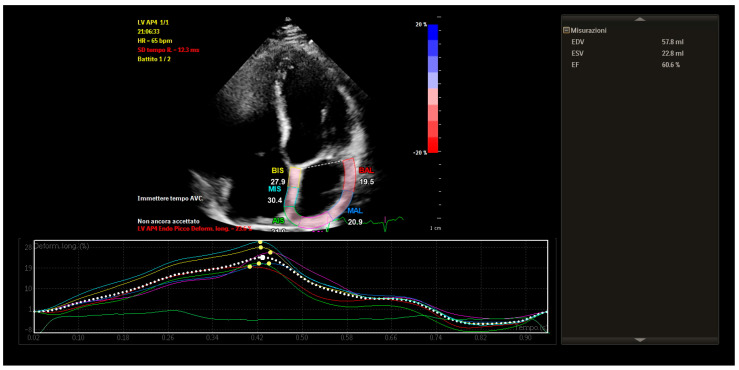
Speckle—tracking echocardiography showing mildly reduced atrial strain with preserved mechanical synchrony.

These findings indicated that, although some subclinical impairment of atrial compliance was present, atrial contractile function was globally coordinated and electromechanically integrated (Figure 4).

**Figure 5:**
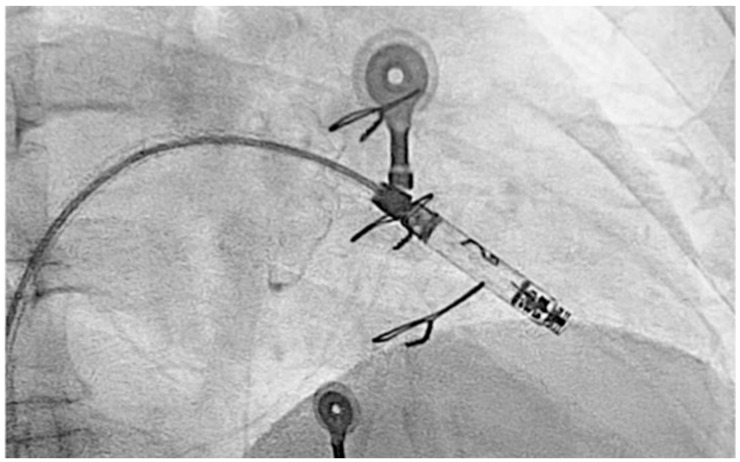
Fluoroscopic image showing the deployment of a leadless ventricular pacemaker in the right ventricle via femoral venous access. The delivery catheter and fixation tines are visible, ensuring secure attachment to the endocardial surface.

Due to the recurrence of conduction disturbances and syncope, a leadless ventricular pacemaker (Aveir, Abbott, IL, USA) was implanted (Figure 5), and beta-blocker therapy was continued for rate control.

## 3. Discussion

This case illustrates the relevance of atrioventricular synchrony, which can be compromised by surgical interventions near the conduction system. Our findings of preserved intra-atrial conduction and mechanical synchrony provide evidence of maintained electromechanical integration, supporting a non-ablation approach to rhythm management.

The mitral annuloplasty may have contributed to the development of AV block, though the latency of onset complicates definitive attribution due to the anatomical proximity of the annuloplasty ring to the AV node and the bundle of His [12]. The pathophysiological mechanisms by which mitral annuloplasty can lead to AV block are primarily related to the anatomical and functional relationship between the mitral annulus and the cardiac conduction system [13]. The AV node and the bundle of His are located in the membranous septum, which lies in close proximity to the anteroseptal portion of the mitral annulus—particularly near the fibrous trigone and the aortomitral continuity. During mitral annuloplasty, especially when sutures or prosthetic rings are placed too medially or too deeply, and depending on anatomical variations in aortic root rotation, mechanical trauma can occur to the conduction tissues [14]. This trauma may disrupt normal electrical conduction either immediately or over time.

In addition to direct mechanical injury, ischemic insult to the conduction system may result from compromised blood supply, particularly from branches of the atrioventricular nodal artery, which can be distorted or compressed during ring implantation. Moreover, the inflammatory response that follows surgical manipulation can lead to edema and local cytokine release, which may transiently impair conduction. In the longer term, healing processes can result in fibrosis and scarring around the conduction tissue, leading to persistent AV block [15]. Furthermore, annular remodeling caused by the rigid or semi-rigid prosthetic ring can alter the geometry and tension of adjacent tissues, possibly exacerbating injury or impeding electrical propagation. These mechanisms collectively explain how a structurally corrective procedure can paradoxically lead to conduction system dysfunction. Importantly, in our case, comprehensive atrial assessment revealed preserved atrial function. In this case, the normal PA-TDI interval and preserved mechanical synchrony on strain imaging suggested intact intra-atrial conduction, supporting the view that the AV block originated at the nodal level rather than reflecting widespread atrial conduction disease. This distinction supported a conservative therapeutic strategy and discouraged unnecessary atrial ablation. The long interval between surgery and the onset of symptoms suggests that progressive age-related fibrosis or chronic mechanical stress may have acted in concert with the initial surgical substrate to provoke late-onset conduction disease.

Regarding the management of atrial fibrillation, while pulmonary vein isolation or posterior wall ablation was initially considered, echocardiographic findings argued against this approach. LA strain imaging provided supportive information on the functional state of the atrial myocardium. While it did not directly guide device implantation, it contributed to the decision to defer ablation. Its role in evaluating atrial mechanics and remodeling is increasingly recognized and has been correlated with both arrhythmic burden and procedural outcomes [16]. In this case, preserved strain and synchrony suggested a relatively healthy atrial substrate, reducing the indication for substrate modification via ablation [17]. Additionally, the patient’s history of hiatal hernia and gastroesophageal reflux suggested a possible vagally mediated gastric trigger for the atrial arrhythmia, further reinforcing the decision to avoid an invasive approach.

The decision to implant a leadless ventricular pacemaker reflected the need for bradyarrhythmia protection without exposing the patient to the risks of transvenous leads. While current studies offer preliminary support for the safety and efficacy of leadless systems, particularly in patients with isolated AV block [18], the evidence base remains limited and evolving. Although cardiac resynchronization therapy (CRT) was not indicated in this case due to preserved ventricular function and absence of bundle branch block, the anatomical complexity imposed by previous valvular surgery would pose challenges for standard CRT lead placement. The choice of a modular leadless pacing system allows for future incorporation of an atrial component, facilitating device-based AV synchrony should it become clinically necessary [19]. Such an approach represents a physiological alternative to conventional CRT in anatomically complex patients. Moreover, patient preference for a device minimizing venous access and maximizing future upgrade options played a significant role in the final decision. This option provides both clinical flexibility and the potential for improved hemodynamic performance over time.

Finally, this case also illustrates the value of a multimodal, non-invasive imaging approach in tailoring therapy in complex post-surgical patients. As highlighted in the recent literature, functional markers such as strain imaging can complement traditional electrophysiological parameters in refining risk stratification and guiding treatment [20].

## 4. Conclusions

In patients with surgically corrected congenital heart disease, late-onset conduction disturbances may reflect the long-term impact of anatomical interventions near critical conduction tissues. In this case, AV block may have been influenced by anatomical alterations related to mitral annuloplasty, though the precise etiology remains multifactorial and uncertain. Echocardiographic parameters, including preserved left atrial volume, normal intra-atrial conduction time, and synchronized atrial strain, were instrumental in guiding a non-ablative management strategy focused on pacing and medical therapy. Left atrial strain imaging proved essential in assessing atrial integrity and informing clinical decisions. This case also underscores the imperative for long-term arrhythmia surveillance in patients undergoing surgical correction of congenital heart disease, particularly those involving mitral or atrial structural interventions.

Furthermore, these findings should be interpreted cautiously, and validation through prospective studies and larger patient cohorts remains necessary.

## Data Availability

Data are available from the corresponding author on reasonable request.

## Abbreviations

The following abbreviations are used in this manuscript:

AV	Atrioventricular
AF	Atrial Fibrillation
TDI	Tissue Doppler Imaging
STE	Speckle-Tracking Echocardiography
LA	Left Atrial
